# Advanced Research in Neuroprotection

**DOI:** 10.3390/biomedicines14020450

**Published:** 2026-02-17

**Authors:** Christos Bakirtzis, Evangelia Kesidou

**Affiliations:** 1 Second Department of Neurology, Aristotle University of Thessaloniki, 54124 Thessaloniki, Greece; 2 Laboratory of Physiology, Faculty of Medicine, Aristotle University of Thessaloniki, 54124 Thessaloniki, Greece

In recent years, neurological research has focused on understudied neurological diseases, uncovering pathophysiological mechanisms and new therapeutic avenues. Inflammatory, immune-mediated diseases of the central nervous system, such as multiple sclerosis and neuromyelitis optica spectrum disorder, are now treated with highly efficacious agents, preventing further disability accumulation [1,2]. With regard to neurodegenerative diseases such as Alzheimer’s disease, synucleinopathies, tauopathies, and motor neuron diseases, significant advances have been made in understanding their genetic susceptibility and potential pathogenic mechanisms [3]. The implementation of novel techniques has enabled the earlier detection of the underlying neurodegenerative processes, and several promising biomarkers for disease monitoring are currently under evaluation for use in everyday clinical practice [4]. Additionally, novel technologies, such as wearables and artificial intelligence, genomic studies, and in-depth research of biofluids, may enable clinicians to detect people at risk early enough, in order to apply interventions at the pre-clinical stages of neurodegenerative disorders [5,6].

Despite these advances, additional research is still needed to explore strategies for repairing neural injury; both neural repair and the reversal of disability remain major unmet goals in everyday clinical practice. The therapeutic armamentarium available to neurologists currently lacks agents capable of preventing further neuronal damage. As a result, clinical management of individuals with neurological disorders is largely limited to controlling comorbid metabolic conditions and promoting a healthy lifestyle [7]. Furthermore, modulation of neuronal plasticity is achieved primarily through physical and cognitive training [8,9], while partial symptom relief may be obtained through agents for symptomatic treatment. Ongoing research aims to provide insight into the mechanisms of neuronal repair, with a focus on identifying potential therapeutic targets to counteract neurodegenerative and age-related processes of the nervous system [10].

In this context, the present Special Issue brings together research focused on the mechanisms of neuronal repair and neuroprotection, along with potential therapeutic interventions. In this Special Issue, we include two studies in animal models that investigate the use of thiamine pyrophosphate as an antioxidant and anti-inflammatory agent in neural damage [11,12]. Furthermore, two studies provide insights into the minimization of neuronal damage of cerebral ischemia by studying the impact of sildenafil and a selenium-derived protein accordingly [13,14]. In another study, deep learning and advanced modelling approaches were applied in a dataset of people with glioblastoma, in order to identify HLA alleles that may be involved in the tumor cell response to immunotherapeutic interventions [15]. Finally, two comprehensive reviews are included in this Special Issue. The first one explores microenvironmental factors affecting the bioavailability of agents used in neurodegenerative diseases and proposes novel drug delivery systems via nanoparticles [16]. A review from the ECF Young Investigators/Fellows Initiative presents novel advances in detecting and measuring remyelination and repair processes in multiple sclerosis [17]. The study of these processes may help to detect novel agents for the treatment of aspects of the disease that remain currently untreated.

Overall, this Special Issue provides insights into current research focused on neuroprotection. This field is rapidly expanding; nevertheless, further studies are needed to elucidate the underlying mechanisms of neural damage and repair. It is hoped that, in the future, novel agents will be tested as candidates for ameliorating what is currently considered irreversible neuronal damage in neurodegenerative disorders. Therefore, we have now launched a second edition of this Special Issue, in order to provide researchers a platform for the presentation of novel, relevant research.
